# Application of Convolutional Neural Network (CNN) to Recognize Ship Structures

**DOI:** 10.3390/s22103824

**Published:** 2022-05-18

**Authors:** Jae-Jun Lim, Dae-Won Kim, Woon-Hee Hong, Min Kim, Dong-Hoon Lee, Sun-Young Kim, Jae-Hoon Jeong

**Affiliations:** 1 The Department of Control and Instrumentation Engineering, Pukyong National University, Busan 48513, Korea; jjl1003@pukyong.ac.kr; 2 The School of Interdisciplinary Management, Ulsan National Institute of Science and Technology, Ulsan 44919, Korea; domokim2@unist.ac.kr; 3 The Department of Radio Engineering, Korea Maritime & Ocean University, Busan 49112, Korea; 4 The Department of Energy Transport System Engineering, Major of Mechanical System Engineering, Pukyong National University, Busan 48513, Korea; 5 The School of Mechanical Engineering, Kunsan National University, Gunsan 54150, Korea; 6 The School of IT, Information and Control Engineering, Kunsan National University, Gunsan 54150, Korea

**Keywords:** convolutional neural network (CNN), recognize ship structures, mask R-CNN, faster R-CNN

## Abstract

The purpose of this paper is to study the recognition of ships and their structures to improve the safety of drone operations engaged in shore-to-ship drone delivery service. This study has developed a system that can distinguish between ships and their structures by using a convolutional neural network (CNN). First, the dataset of the Marine Traffic Management Net is described and CNN’s object sensing based on the Detectron2 platform is discussed. There will also be a description of the experiment and performance. In addition, this study has been conducted based on actual drone delivery operations—the first air delivery service by drones in Korea.

## 1. Introduction

The COVID-19 pandemic has dramatically changed our daily lives. To avoid personal contact and prevent the spreading of the virus, people had to work or learn from their homes through online platforms. Shops and restaurants had to find ways to survive through online sales and home delivery services. This trend has brought significant changes in the maritime sector as well. Seafarers were not permitted to go ashore due to restrictions imposed by local governments. Global port congestions caused ships to stay longer in the anchorage areas, waiting for the berth to be available to load/unload the cargo. This has resulted in seafarers’ accumulated fatigue and stress [1]. It has also brought an urgent need for a new maritime delivery service that connects land and sea in a non-face-to-face manner.

To respond to this need, the Busan Regional Aviation Administration has issued a business license to Marine Drone Tech Inc., launching a first-ever commercial drone delivery service in Korea for seafarers and ships visiting Busan Port. It has shown steady progress—about 300 deliveries as of 21 December 2021. Accurate ship information is essential to ensure safe and efficient maritime deliveries.

During the maritime delivery, we use the Automatic Identification System (AIS) to collect information on ships, such as names and locations [2,3,4,5,6]. AIS is an automatic ship identification system that broadcasts ship information through VHF-based AIS terminals. To facilitate maritime traffic control, the system automatically transmits and receives static information (ship’s name, MMSI, type, and size), dynamic information (ship’s position, speed, heading, and course), and navigation information (ship’s outport, next port, and expected arrival time) between land and ship control offices and between ships and ships. However, since the AIS checks the location of ships in a non-visual way, it does not have the ability to determine their appearance and structure on the deck, which may be a risk factor for drones’ flying and landing [7,8,9]. This is an uncertainty factor in the maritime delivery service using drones, which does not guarantee safety. To overcome this problem, it is common to establish a systematic flight plan by searching the Internet and collecting detailed ship information in advance, such as information on the appearance, delivery point, and structure with a collision risk. In addition, we visually check the vessel in actual operation through a telescope and compare the differences with the information collected in advance to finalize the flight plan. However, the above method is time-consuming and has limitations—it can be operated only by personnel with abundant knowledge of the sea and ships, and it is difficult to check the situation and structure on the deck accurately. This is a fatal problem that lacks safety and usefulness to activate the maritime delivery service.

To solve this problem, there is an autonomous system that can directly check and identify moving objects. The autonomous system is divided into land and sea. In the case of land, the system is widely used as an element of technology for autonomous navigation, which identifies pedestrians, vehicles, and traffic lights. However, in the case of the sea, little research has been made on the system. In particular, there are few datasets built based on images obtained from the flight altitude of flying objects [4]. Therefore, it is necessary to establish a dataset for the autonomous system of the entire process of the maritime delivery service, automatically identify ships using visual sensors, and study the recognition of ship structures with collision risks. 

Therefore, this paper uses a visual sensor to build a dataset based on the images obtained from the flight altitude of flying objects and create a learning model to verify whether the system applies to the autonomous process of future maritime delivery. This is expected to contribute to the vitalization of maritime delivery service and the autonomy of the entire process. The paper also conducted the following two research activities: 

First, we conducted the sea delivery service 300 times for ships anchored in the Busan Port [2,3,4,5,6]. We also created the marine traffic management (MTMnet) dataset by extracting images from videos taken between the maritime delivery services. The dataset has the following characteristics:

(1) Since we extracted images from ships anchored in Busan Port for the past year, the dataset provides information on the appearance of the ships (painting and structure) that matches operated ships and includes up-to-date visual information about the ships.
(2) Since the dataset is extracted from images taken during the maritime delivery service, it includes visual information taken from various angles and distances from the center of ships.

These characteristics will be updated through AI learning, which will be helpful for more reliable and specific object recognition with diversified visual information. 

Second, we select an optimal AI model that can simultaneously perform ship classification and structure recognition using visual sensors and repeatedly learned it using the MTMnet dataset. We compared and analyzed each model using the Detectron2 platform to apply diverse AI models [10,11,12,13,14,15].

Based on the results, we evaluated the validity and performance of the dataset and learning model presented in this paper. We also considered the applicability for the development of autonomous maritime delivery services in the future.

## 2. Dataset of Marine Traffic Management Net (MTMnet)

With the recent development of information and communication technology and artificial intelligence technology, various means of transportation such as airplanes, automobiles, and ships are applying fully automated transportation systems. Nevertheless, a study on the visual interpretation of ships is necessary because the maritime industry is falling behind in this application, unlike other land sectors [9]. AIS radars and SAR are usually used for obtaining information on ships, but they cannot secure accurate visual cues in real-time, such as the size and structure of ships [2,3,4,5,6,7,8,9]. 

For the acquisition of visual information on ships in the sea, there is a limitation in that drones are subject to the Beyond Visual Line of Sight (BVLOS). Therefore, most studies on the visual interpretation of ships focus on public datasets (MODD, SMD, IPATCH, SAGULL, and VOC2007) [9,16] and image searches on Google [7,8]. It is also known that there are no studies with datasets obtained through the direct imaging of drones. This is advantageous in terms of the ease of collection and the time needed to construct datasets. 

**Figure 1:**
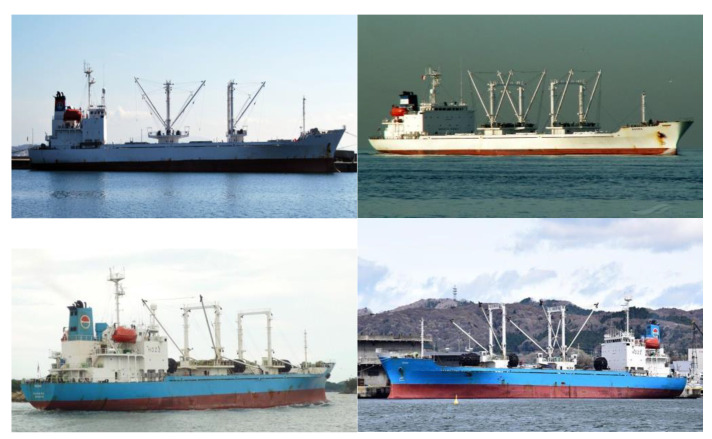
Search Results of SUAH_Reefer on Google.

According to Figure 1, most of the data are taken from the sea face rather than aerial photography data reflecting the drones’ point of view. 

This provides visual cues that are too limited to recognize three-dimensional ships and precise structures. To classify ships with the cameras of drones performing the maritime delivery service and precisely recognize structures with collision risks, we need to build a dataset with images taken from more diverse angles and distances. This necessitates data collection and continuous management through actual drone flights to study the visual interpretation of ships.

**Figure 2:**
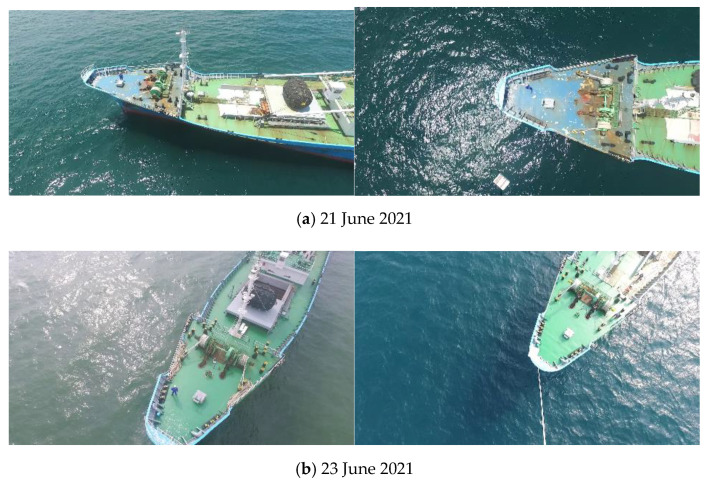
Case of SUAH_Reefer Paint Color Change in the Southern Outer Anchorage of Busan Port.

Figure 2 shows the change in the visual information of the ships as the paint color of the same ship is partially changed within two days. It is expected that this makes it difficult to predict the appearance of actually-operating ships and also adversely affects the AI learning performance.

**Figure 3:**
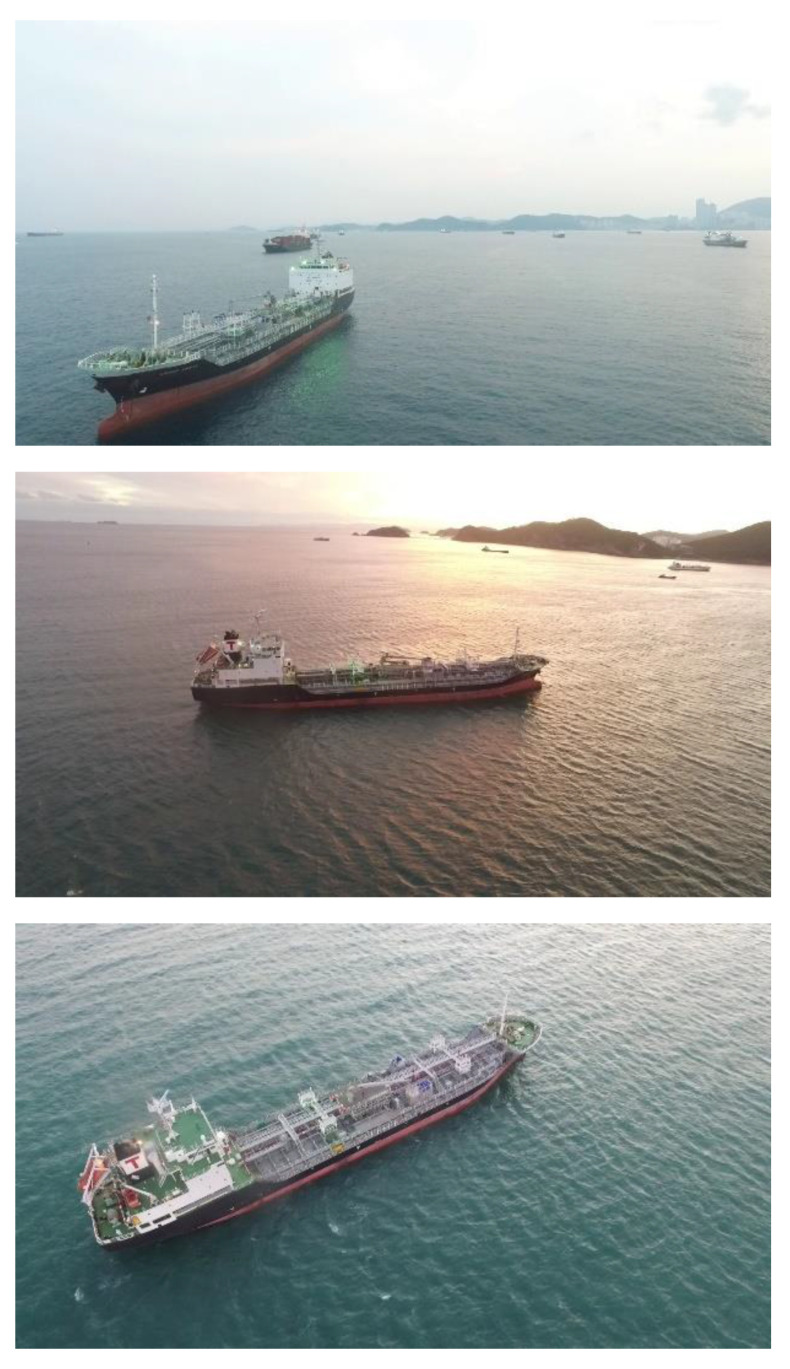

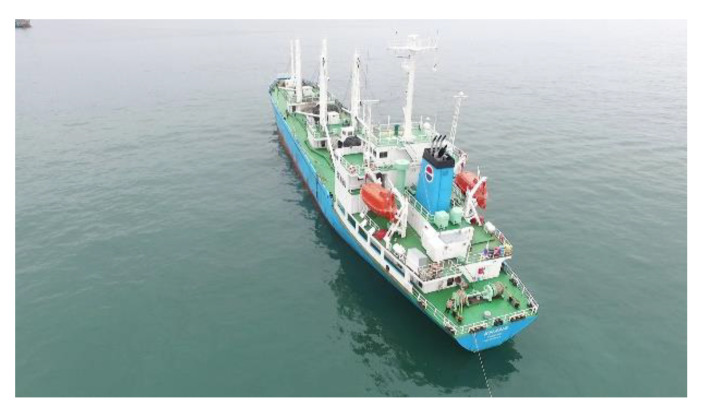
Ship Images Included in the MTMnet Dataset.

Figure 3 shows some of the ship images included in the MTMnet dataset. These images are extracted from videos taken during the maritime delivery service. The images are taken at various angles and distances compared to the existing ship images in Figure 1 and include detailed appearance information of the ships in actual operation. Therefore, it is expected that the images are effective for ship classification and precise structure recognition.

However, the existing research points out that problems arise when using aerial image-based datasets for AI learning. Aerial photographed images may include various sizes of ships, from small to large, for the learning. Due to various topographical features or irregular objects such as garbage floating in the sea and changes in the surrounding background by weather changes, this may lead to the deterioration of the performance of ship classification and accurate structure recognition. To resolve this problem, various studies suggest adding preprocessing to improve the performance, such as the application of data argumentation or ensemble algorithms to the dataset for AI learning and the background invariant method to prevent performance degradation due to changes in the surrounding background [17,18,19]. In addition, it is considered that we need to collect and learn data reflecting various cases such as weather conditions and irregular-sized objects by continuing to fly drones to improve learning performance. 

**Figure 4:**
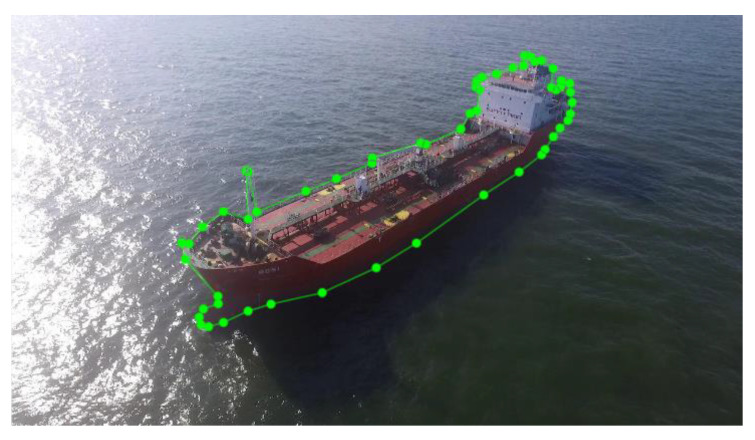
Polygonal Annotation.

In this paper, we extracted images from video data acquired by performing the maritime delivery service about 300 times from 2021 for ships anchored in the Southern Outer Anchorage of Busan Port [2,3,4,5,6] and constructed the dataset of MTMnet. The dataset consists of the latest visual data of ships anchored in Busan, which have been obtained by the drones performing the maritime delivery service. This paper also performed data labeling as follows to identify information on structures that are likely to present risks of collision during the maritime delivery service. First, as shown in Figure 4, we performed polygonal annotation with green dots and lines on a total of 1536 files.

Second, we converted the data annotated with the LabelMe program consisting of the labelme2coco algorithm to the COCO dataset format. 

For detection, classification, and segmentation in the COCO dataset format, we constructed the necessary information such as the coordinates of the bounding box and the segmentation mask pixels in JSON. 

Through AI learning with the dataset, we identified ships and materialized information on ship structures, which are risk factors for the maritime delivery service.

## 3. CNN Object Detection Based on Detectron2 Platform

The existing object recognition models such as YOLO, Fast R-CNN, and Faster R-CCN usually use the method to classify recognized objects in the bounding box. However, to perform a safe maritime delivery service, the model needs to not only classify ships but also detect objects and structures with collision risks at the pixel level. This requires the additional creation of a segmentation mask for each instance in the image. The mask RCNN is a representative model that implements an instance segmentation function capable of classification and localization [20].

**Figure 5:**
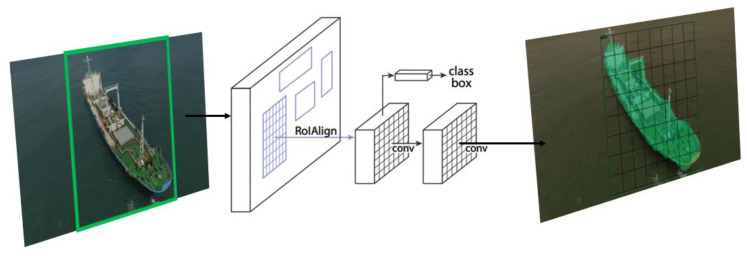
Mask R-CNN Architecture.

The model shown in Figure 5 is an instance segmentation model using the Mask R-CNN to realize detection as well as segmentation. Mask R-CNN is a model that adds one additional step to predicting segmentation masks for each Region of Interest (Rol) of Faster-CNN, which is used as an existing-object detector. If used by Faster R-CNN for classification, such a small difference does not yield a big result. However, in the case of Mask R-CNN, which performs prediction on a pixel-by-pixel basis, even the tiniest difference can lead to large changes in the results. To solve this problem, we used the RoIAlign layer instead of the RoIPool layer, which performs harsh quantization. We compared all three methods—RoIPool, RoIWarp, and RoIAlign—and obtained the best results from RolAlign [20,21,22,23].

MASK R-CNN has a two-stage structure for object recognition. First, the model implements the region proposal network (RPN). Second, it performs a binary mask for each predicted class, box offset, and RoI. Additionally, the loss function applies multi-task loss independently to the ROI. For semantic segmentation, the amount of computation is high because the class and mask must be predicted for each pixel. Therefore, some studies separately predicted the class and mask of the Mask R-CNN to reduce the amount of computation [20,21,22,23].

In this paper, we performed class prediction and mask prediction at the same time. In consideration of stable operation and the reduction of drone flight time, we do not perform calculations on a single board computer (Raspberry Pi and Jetson) but proceed with desktop-based AI learning to perform calculations on the land. This is a concept in which the image data obtained by the camera mounted on the drone communicate with a desktop on the ground learned through a single board computer to allow for ship classification and structure recognition. Additionally, we used the Detectron2 platform for the comparative analysis of more diverse models. Detectron2 is a Facebook AI Research (FAIR) software system that implements advanced object detection algorithms including Faster R-CNN, Mask R-CNN, RetinaNet, and Densepose. Detectron2 is the latest version of Detectron and is optimized with PyTorch. It has the advantages of flexibility and extensibility, so it is widely used in FAIR [10,11,12].

In this paper, we compared and analyzed the object recognition performance of Faster R-CNN and Mask R-CNN using the Dectectron2 platform on our custom-made MTMnet dataset. To increase the importance of the AI to accurately recognize ship structures, which is the main purpose of this research, we compared and analyzed the object recognition performance by allowing the AI to learn additional networks of R50-C4. To compare and analyze the performance of 3x, R50-DC5 3x, and R50-FPN 3x for Mask R-CNN, we evaluated the performance with average precision (AP) based on the intersection over union (IOU). IOU stands for Overlapping Region/Combined Region, meaning the overlapping degree of boxes. If the IOU overlaps by 0.5 at AP 50, we evaluate it as correct. We also evaluated it as correct when the IOU overlaps by 0.75 at AP 75. AP means the average of AP50, AP55, AP60…AP95 [24].

## 4. Experimental Results and Performance Evaluation

In this experiment, we compared the object recognition rate of ships and structures by comparing Faster R-CNN and Mask R-CNN. We also confirmed that Mask R-CNN shows more excellent recognition performance than Faster R-CNN.

### 4.1. Faster R-CNN

We prepared a set of ship images in a certain size and classified the label as “ship” to increase the training accuracy of the Faster R-CNN model. We imported the data into Python by randomly mixing the MTMent dataset and dividing it into 70% for training, 20% for validation, and 10% for evaluation. We generated a Python code in JupyterLab and added Numpy, Matplotlib, etc. for machine-learning packages. The Faster R-CNN model was trained with the R50 FPN 3x backbone network, and the accuracy and results of the model are shown in Table 1.

Table 1 shows that the Faster R-CNN model has a relatively good performance (an accuracy of 0.901) when the AP 50 IoU (intersection over union) is 0.5, but it indicates that the accuracy is lowered to 0.516 when the AP 75 IoU is 0.75 or more.

**Figure 6:**
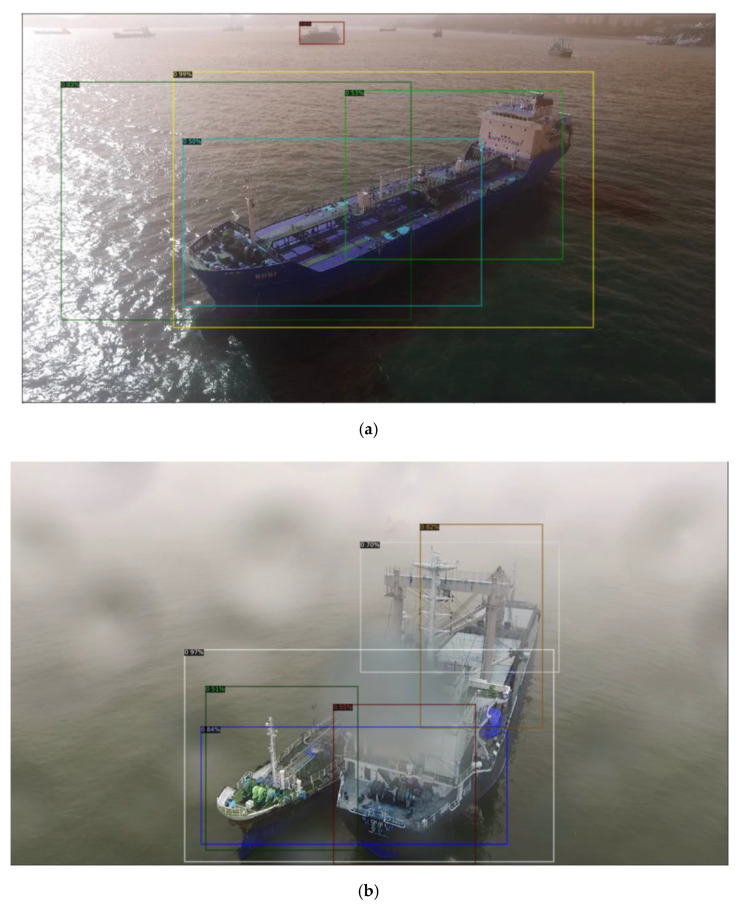

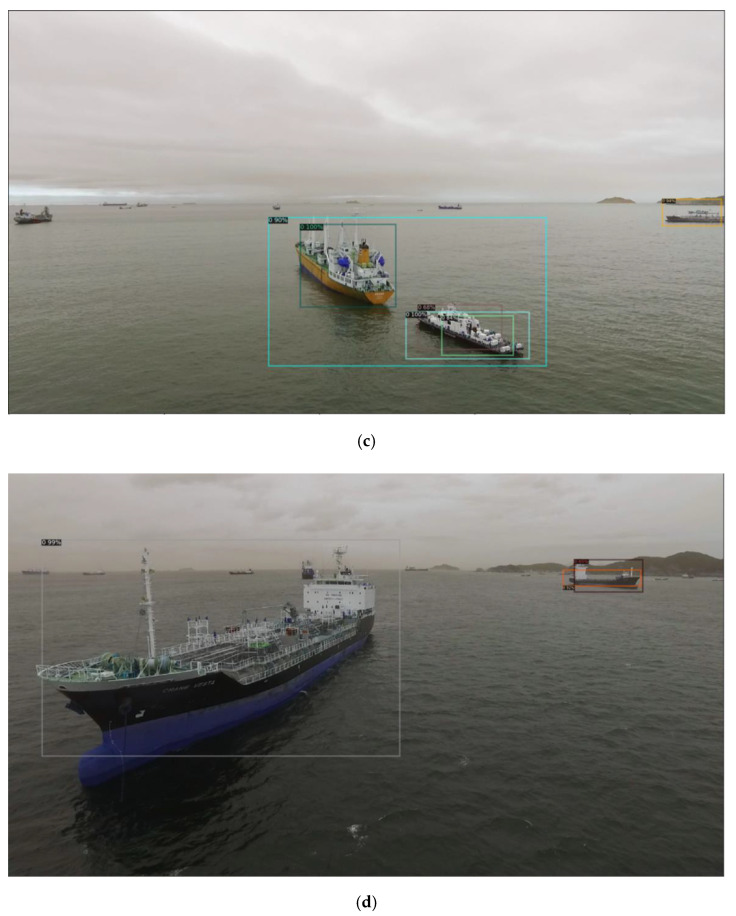

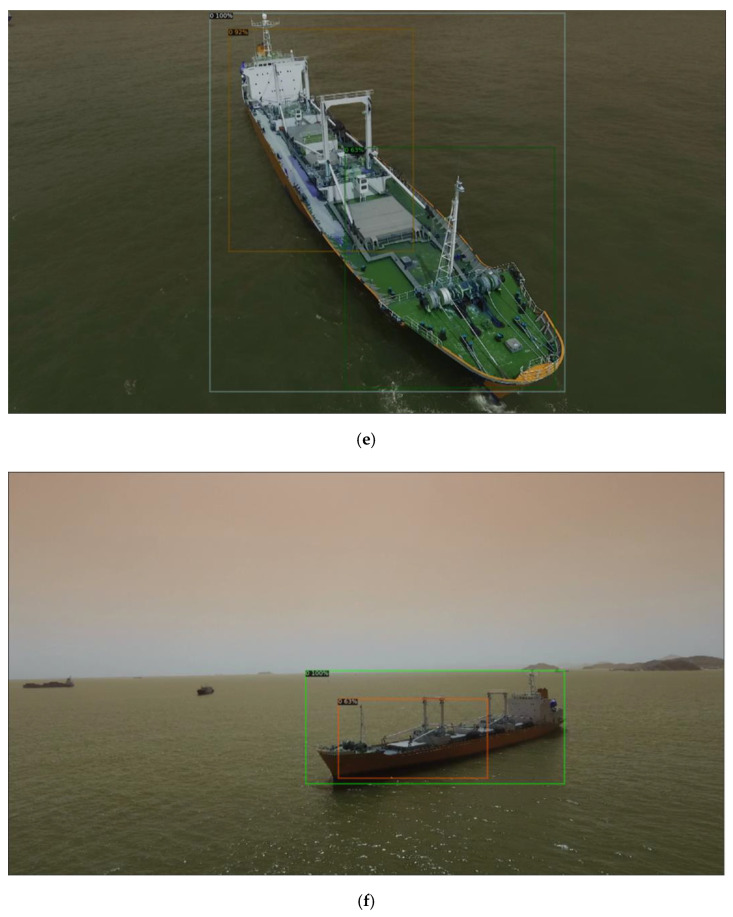
Test Results of Faster RCNN.

Figure 6 shows the classification results of ships classified by the Faster R-CNN model. Looking at the results of the following images, the accuracy of the classification of the bounding box is high in the case of a relatively simple ship, as shown in Figure 6d. However, in the case of the ship shown in Figure 6a–c,e,f, recognition errors occurred because the ship structures were classified as separate ships.

### 4.2. Mask R-CNN Bounding Box Results

**Figure 7:**
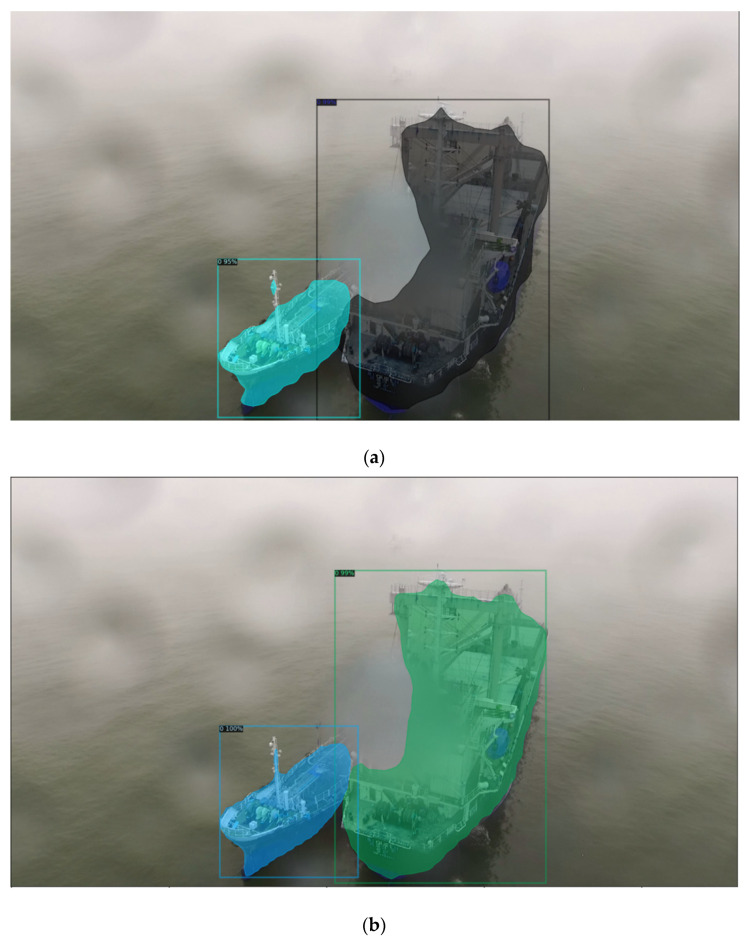

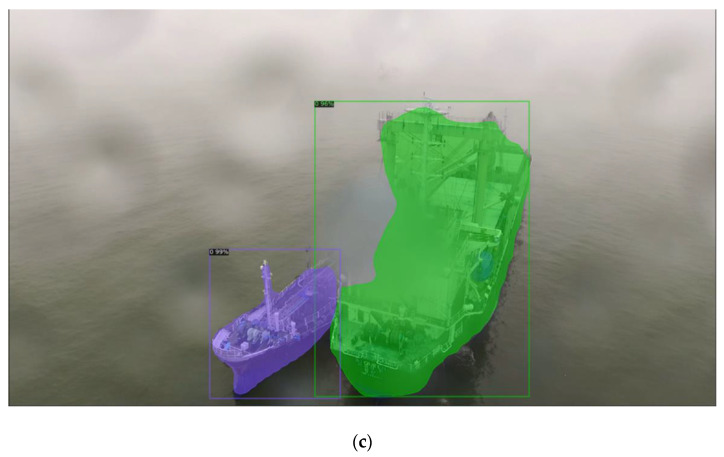
Comparison of the Bounding Model Test Results for the Mask R-CNN Model. (**a**) Bounding Box Result of R50-C4 3x; (**b**) Bounding Box Result of R50-DC5 3x; (**c**) Bounding Box Result of R50-FPN 3x.

The experiment in Table 2 shows the results obtained by comparing the performance of the Bounding Box by additionally learning the R50-C4 3x, R50-DC5 3x, and R50-FPN 3x backbone networks of Mask R-CNN. The ship image in Figure 7 is an image taken by the drone performing a maritime delivery service on a rainy day. It is difficult to identify the ship because water droplets rain on the drone and block its camera. Nevertheless, we could confirm that Mask R-CNN, which has undergone additional training, showed higher performance than Faster R-CNN. However, it is not easy to use drones in rainy conditions. The video data are a case in which it was not raining when the drone took off, but it suddenly rained during the process of approaching the ship. This is a very small part of the dataset, but it is judged that the drone may not recognize the ship depending on the degree of water drops, although the drone accurately recognizes the ship on a clear day. Nevertheless, the instance segmentation model Mask R-CNN, which can classify objects at the pixel level, shows relatively better performance than Faster R-CNN.

### 4.3. Mask R-CNN Segmentation Results

**Figure 8:**
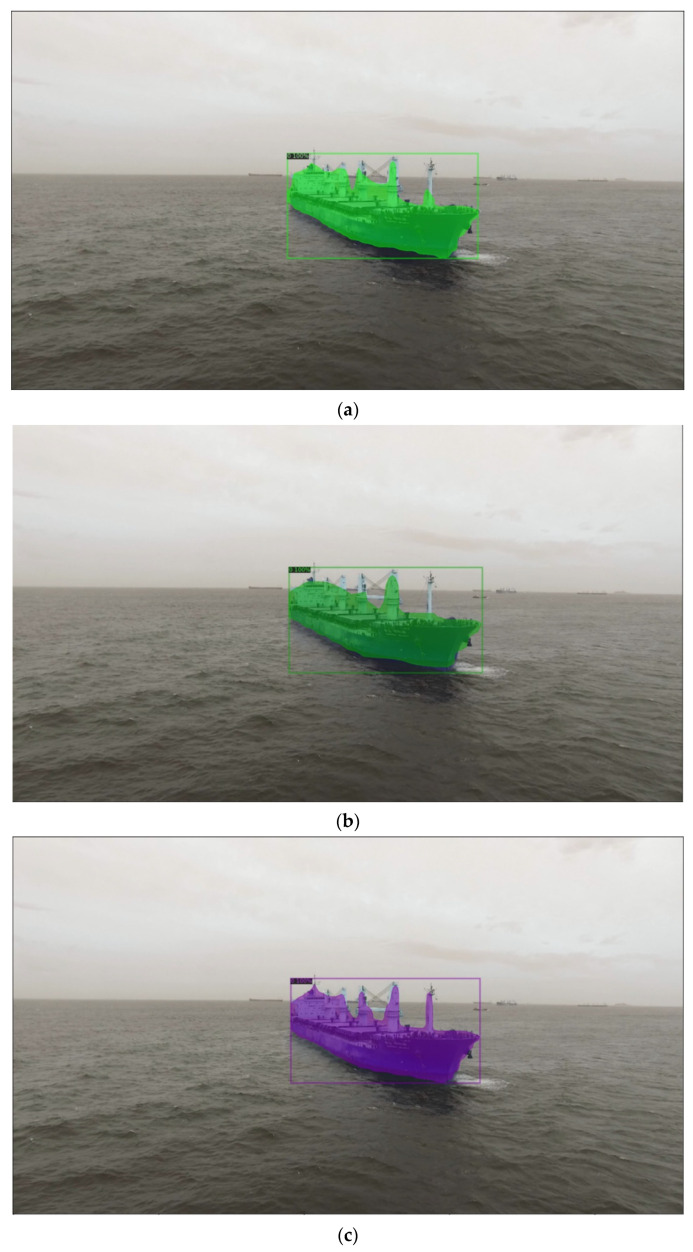
Comparison of the Segmentation Test Results of the Mask R-CNN Model. (**a**) Segmentation Result R50-C4 3x; (**b**) Segmentation Result of R50-DC5 3x; (**c**) Segmentation Result of R50-FPN 3x.

The results of the experiment in Table 3 show the comparison of segmentation performance by additionally training the backbone networks of R50-C4 3x, R50-DC5 3x, and R50-FPN 3x for Mask R-CNN. All three backbone networks show relatively high performance, and R50-FPN 3x 5000 has the highest result. Comparing images (Figure 8a–c) shown in Figure 8, the resulting images using the R50-C4 3x backbone network recognized the shape of the ships and large structures. However, this had a limitation in that many of the thin structures of the ships were not recognized. The resulting images using the R 50-DC5 3x backbone network (Figure 8b) showed improved performance compared to (Figure 8a). The resulting images using the R50-FPN 3x backbone network (Figure 8c) were able to recognize the structures that were not recognized in (Figure 8a,b), and it has a more precise recognition rate of distinguishing between ships and structures.

However, in the case of segmentation, it is considered that it is more suitable to utilize the data learned on the desktop on the ground rather than the single-board computer with a relatively complex operation. It is judged that the ship classification and precise structure recognition will be well implemented without additional learning, except for special cases such as rainy situations. We used the trained model to further test the video file to confirm its utility.

**Figure 9:**
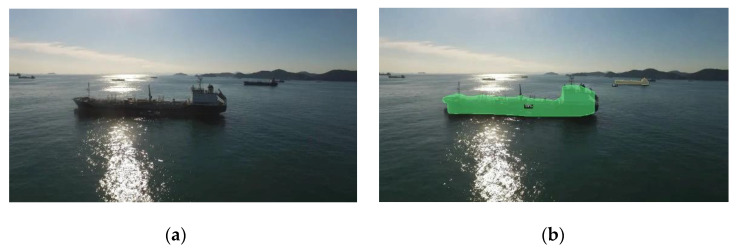
Comparison Result of the Original Video (**a**) and Ship Detection Using the Trained Mask R-CNN Model (**b**) in the Same Frame Screenshot.

We detected ships using the Mask R-CNN model trained for testing on video files and compared the original video frame and the output video frame. Figure 9 shows each screenshot for the video image. The trained Mask R-CNN model can detect almost all ships in the original image. In a very short time, it provides segmented ship and structure information. The hardware specification for the test is based on CPU: Intel^®^ Xeon^®^ CPU @ 2.00 GHz and RAM: 13 GB of the Colab platform. A video file takes about 0.09 s to 0.11 s to generate segmentation information. In other words, the model can recognize approximately 10 frames per second, thereby generating information in a very short time. However, the model often does not recognize small-sized ships in the current video files, so we need to conduct future research to resolve this problem. 

### 4.4. Mask R-CNN Segmentation Accuracy and Loss Curve

As a result of comparing the learning performance of Faster R-CNN and Mask R-CNN, we confirmed that the Mask R-CNN model showed better learning performance. To accurately recognize the ship structures that may become an obstacle to drones flying during maritime delivery service, we analyzed the curves of the accuracy and loss of the Mask R-CNN segmentation.

**Figure 10:**
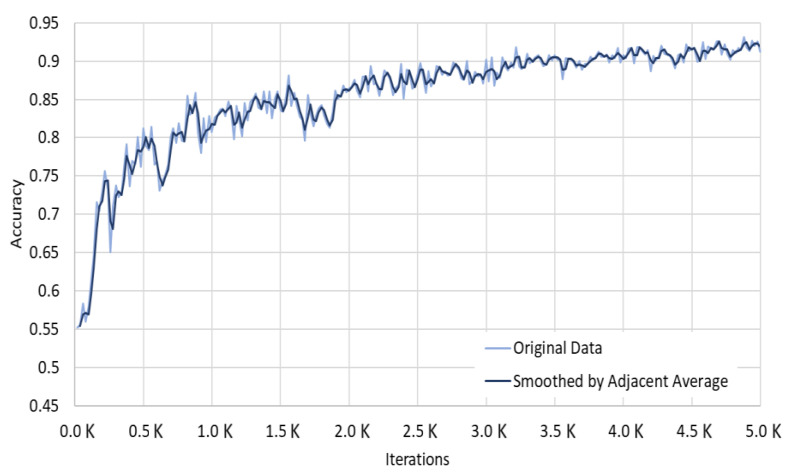
MASK R-CNN Segmentation Accuracy Curve (source: TensorBoard Image).

**Figure 11:**
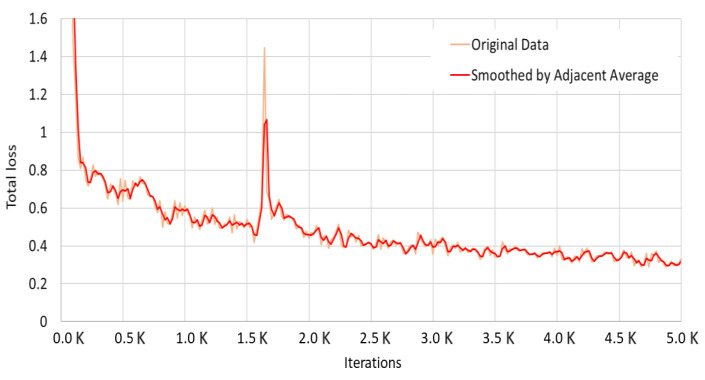
MASK R-CNN Segmentation Loss Curve (source: TensorBoard Image).

As shown in Figure 10, Mask R-CNN showed an accuracy of about 90% starting with 3500 learning experiences, which converged to about 95% with additional learning up to 5000 times. We checked the loss curve of the Detectron2 model for additional performance evaluation and used Google Colab GPU for 5000 iterations. We tracked the total loss value using TensorFlow and confirmed the curve shown in Figure 11. We could confirm that the total loss value decreases as the learning iterations increase and finally reaches about 0.3.

## 5. Conclusions

In this paper, we built our MTMnet dataset by using images taken by drones performing maritime delivery services. This dataset constitutes up-to-date external images of ships anchored in the Southern Outer Anchorage of Busan Port and has the characteristics of aerial photography taken by drones rather than pictures taken on the sea surface. In addition, we successfully performed the identification of ships and their structures by applying various backbone networks of Faster R-CNN and Mask R-CNN through the Detectron2 object detection platform using the MTMnet dataset. Simply applied Faster R-CNN has high accuracy in the classification of bounding boxes of relatively simple ships. However, it yields errors in the classification of ships with complex structures and is incapable of recognizing specific structures. 

To resolve this problem, we applied an R50-FPN 3x backbone network to Mask R-CNN, and we confirmed that this combination can precisely recognize the complex structures. We strongly believe that this finding will be a foundation for the stability and automation of marine delivery services using drones. It is considered that the number of datasets and the amount of research related to artificial intelligence in the maritime field are far behind those of land. Although we were able to obtain relatively satisfactory research results through this study, there are many thin structures in ships that are difficult to recognize by AI, such as wires. In this study, we confirmed that raindrops on the camera lenses of flying drones become an obstacle to object recognition. This case may be extremely rare due to the nature of the operation of drones, but it may occur during the operation. In this regard, it is considered that we need to collect additional data, perform data augmentation to resolve this obstacle, and verify the model in diverse environmental conditions. Therefore, it is expected that the whole process automation of maritime delivery services using drones will be activated if regular datasets are updated and further studies are performed to recognize more complex ship structures using AI in a maritime environment.

## Figures and Tables

**Table 1:** Comparison of the Faster RCNN Model and Repeated Test Results (Bounding Box).

Model	Iterations	AP	AP50	AP75	APm	APl
R50 FPN 3x	5000	0.482	0.901	0.516	0.309	0.493

**Table 2:** Comparison of Various Models and Repeated Test Results (Bounding Box).

No.	Model	Iterations	AP	AP50	AP75	APm	APl
1	R50-C4 3x	1000 5000	0.811 0.833	0.996 0.998	0.945 0.949	0.748 0.681	0.819 0.846
2	R50-DC5 3x	1000 5000	0.836 0.876	0.992 0.999	0.959 0.966	0.645 0.710	0.847 0.890
3	R50-FPN 3x	1000 5000	0.799 0.874	0.997 0.999	0.964 0.951	0.704 0.758	0.805 0.881

**Table 3:** Comparison of Various Models and Repeated Test Results (Segmentation).

No.	Model	Iterations	AP	AP50	AP75	APm	APl
1	R50-C4 3x	1000 5000	0.724 0.751	0.996 0.998	0.939 0.952	0.549 0.628	0.731 0.753
2	R50-DC5 3x	1000 5000	0.776 0.818	0.992 0.999	0.962 0.969	0.519 0.712	0.786 0.824
3	R50-FPN 3x	1000 5000	0.770 0.834	0.996 0.999	0.956 0.970	0.667 0.747	0.773 0.840

## Data Availability

Not applicable.
